# Exercise and functional foods

**DOI:** 10.1186/1475-2891-5-15

**Published:** 2006-06-05

**Authors:** Wataru Aoi, Yuji Naito, Toshikazu Yoshikawa

**Affiliations:** 1 Research Center for Sports Medicine, Doshisha University, Kyoto 602-8580, Japan; 2 Department of Inflammation and Immunology, Graduate School of Medical Science, Kyoto Prefectural University of Medicine, Kyoto 602-8566, Japan; 3 Department of Medical Proteomics, Graduate School of Medical Science, Kyoto Prefectural University of Medicine, Kyoto 602-8566, Japan

## Abstract

Appropriate nutrition is an essential prerequisite for effective improvement of athletic performance, conditioning, recovery from fatigue after exercise, and avoidance of injury. Nutritional supplements containing carbohydrates, proteins, vitamins, and minerals have been widely used in various sporting fields to provide a boost to the recommended daily allowance. In addition, several natural food components have been found to show physiological effects, and some of them are considered to be useful for promoting exercise performance or for prevention of injury. However, these foods should only be used when there is clear scientific evidence and with understanding of the physiological changes caused by exercise. This article describes various "functional foods" that have been reported to be effective for improving exercise performance or health promotion, along with the relevant physiological changes that occur during exercise.

## Introduction

Appropriate nutrition is essential for the proper performance of exercise. In particular, correct nutrition is critically important for improvement of athletic performance, conditioning, recovery from fatigue after exercise, and avoidance of injury. Although athletes need to eat a well-balanced basic diet, there are several nutritional factors that are difficult to obtain at a sufficient level from a normal diet since athletes require more nutrients than the recommended daily allowances. Thus, nutritional supplements containing carbohydrates, proteins, vitamins, and minerals have been widely used in various sporting fields, partly because these supplements are easily taken before, during, and/or after exercise. Several natural food components have also been shown to exert physiological effects, and some of them are considered to be useful (when ingested at high doses or continuously) for improving athletic performance or for avoiding the disturbance of homeostasis by strenuous exercise. Recently, food components with physiological actions have been called "functional foods" and the effects of such foods have been scientifically investigated. This article introduces some functional foods, including basic nutrients, which have been demonstrated to have a beneficial influence on the physiological changes that occur during exercise.

## Replenishment of Water

Water is the main constituent of the human body, and it plays an essential role in circulatory function, chemical reactions involved in energy metabolism, elimination of waste products, and maintenance of the body temperature and plasma volume. When the body temperature rises due to the intense exercise or a high ambient temperature, sweating occurs in order to radiate heat [1-3], leading to the loss of a large amount of water and electrolytes such as sodium. This loss of body fluid impairs thermoregulation and circulatory system, leading to a decline of athletic performance [4,5]. Therefore, to maintain homeostasis and athletic performance, replenishment of water and electrolytes is essential before and during or after exercise. Generally, it is believed to be useful to drink isotonic fluid that contains electrolytes such sodium, potassium, and chloride at concentrations close to those in body fluids. It has also been suggested that intake of hypotonic fluid may exert a similar or more rapid effect on replenishment of body water [6,7] because it is rapidly absorbed from the small intestine along an electrochemical gradient. Also, the sodium concentration (and the osmolality) of sweat is lower than that of extracellular fluid, so the loss of water with sweating is much greater than the loss of electrolytes, leading to an increase in the plasma osmotic pressure. On the other hand, replenishment of water alone is unlikely to maintain homeostasis of body fluid in prolonged exercise that produces high sweat rates. Taking in only water in prolonged exercise leads to hyponatremia and a decrease in the osmotic pressure of body fluids and inhibit the release of antidiuretic hormone resulting in that water intake is suppressed and the urine output is increased (spontaneous dehydration) [8]. Latzka et al. [9] suggested that during prolonged exercise lasting longer than 90 minutes, fluid drink containing electrolytes and carbohydrate, not water alone, should be considered to provide to sustain carbohydrate oxidation and endurance performance. Furthermore, several studies have suggested that glycerol loading has been advocated as one of methods which prevent high temperature and dehydration in exercise [10,11]. Oral administration of 1.0–1.2 g/kg B.W. glycerol with water temporarily results in an increase of 300–700 ml body fluid [10,11] and improves endurance performance compared with placebo [12-14]. Glycerol acts as an osmolyte in body fluid, which would lead to an elevation of plasma osmolality [10]. Consequently, water reabsorption in the kidney is increased and urine excretion is decreased, which is considered as one of mechanisms of the effect.

## Improvement of Endurance

Energy consumed during exercise is mainly supplied by carbohydrates and lipids, so it is important for improvement of endurance to regulate the metabolism of these two substrates. During endurance exercise, glycogen (an energy substrate for muscle contraction) is gradually depleted, making it difficult to continue exercising. An effective way to improve endurance is to increase the glycogen stores in skeletal muscle and the liver before the commencement of exercise. When tissue glycogen stores are depleted, glycogen synthetase activity is transiently increased, leading to an increase of glycogen storage by conversion from carbohydrates [15,16]. For instance, it has been reported that glycogen stores can be increased by eating a low-carbohydrate diet for 3 days from 6 days prior to competition, followed by a high-carbohydrate diet for the next 3 days, resulting in the storage of 1.5 times more glycogen than normal [17]. If citrate, which inhibits glycolysis, is taken concurrently with a high-carbohydrate diet, glycogen stores will be further increased due to the inhibition of glycolysis [18,19]. It is also important for athletes to replenish the glycogen stores during post-exercise training to provide sufficient energy for the next training session or competition. For rapid replenishment of glycogen stores, a high-carbohydrate diet can be effective [19,20]. Intake of protein along with carbohydrate can be more effective for the rapid replenishment of muscle glycogen after exercise compared with carbohydrate supplements alone [21,22].

When prolonged exercise will be performed, such as a marathon, taking carbohydrates immediately before or during exercise is also an effective method of improving endurance. Under such conditions, it is desirable for the athlete to ingest monosaccharides or oligosaccharides, because these are rapidly absorbed and transported to the peripheral tissues. On the other hand, intake of carbohydrates inhibits the degradation of fat, which is another energy substrate, by stimulating insulin secretion [23,24]. This leads to impairment of energy production via lipid metabolism and accelerates glycolysis as alternate energy production pathway. As a result, the consumption of muscle glycogen will increase, and the intramuscular pH will decrease due to increased lactic acid production, which may lead to impairment of muscle contraction. Therefore, it is necessary to ingest carbohydrates that will not inhibit lipid metabolism. It has been suggested that supplements containing fructose, which cause less stimulation of insulin secretion and are unlikely to inhibit lipolysis, rather than common carbohydrates such as glucose and sucrose, may be better for improving endurance [25]. Furthermore, simultaneous intake of citrate can be expected to promote energy consumption from lipids through inhibition of glycolysis [26]. This will spare glycogen and inhibit lactic acid production, so that the weakening of muscle contraction will be delayed. An amino acid, arginine, has been reported to modulate hormones that control the blood glucose level without inhibiting lipid metabolism, and to delay glycogen depletion during exercise [26,27]. Therefore, intake of both citrate and arginine along with carbohydrates that cause little stimulation of insulin secretion before or during exercise may be an effective way to improve energy metabolism and to supply the optimum energy sources for prolonged exercise.

If there is a shift from predominantly glucose-based energy consumption to lipid-based energy consumption, this may lead to improvement of endurance by maintaining glycogen stores and inhibiting the decrease of intramuscular pH that results from generation of lactate during exercise. Several authors have reported about various factors that can stimulate lipid metabolism, although there is insufficient evidence about their efficacy. Carnitine is an intracellular enzyme that is required for fatty acid transport across the mitochondrial membrane into the mitochondria, and it promotes the β-oxidation of fatty acids [28,29]. Carnitine supplementation is expected to activate lipid metabolism in the skeletal muscles, and to also achieve the sparing of glycogen stores. In persons performing aerobic training, intake of 2–4 g of carnitine before exercise or on a daily basis was reported to increase the maximum oxygen consumption (anaerobic threshold) and also inhibited the accumulation of lactate after exercise [30,31]. The effect of caffeine on endurance has also been studied. Caffeine inhibits phosphotidiesterase by promoting catecholamine release and increases hormone-sensitive lipase (HSL) activity, which leads to an increase of circulating free fatty acids and further improvement of endurance [32,33]. Capsaicin, obtained from hot red peppers, is likely to enhance fat metabolism by altering the balance of lipolytic hormones and promoting fat oxidation in skeletal muscle [34,35].

## Enhancement of Muscle Strength

It is well known that the strength of a muscle is generally proportional to its cross-sectional area, and it is necessary to increase muscle bulk in order to enhance strength. Muscle tissue is mainly composed of proteins (such actin and myosin) and water, and it is important to increase the protein content by modulating protein metabolism when increasing muscle bulk. In other words, muscle bulk and strength can be increased by promoting protein synthesis or by inhibiting protein degradation. Resistance exercise is aimed at increasing muscle bulk, and it enhances the secretion and production of growth hormone and various growth factors [36]. Thus, resistance exercise promotes protein synthesis and an increment of muscle mass more strongly compared with aerobic exercise. In order to maximize the effect of resistance exercise, it is important to maintain the muscular pool and blood levels of various amino acids that are substrates for the synthesis of muscle proteins. For this purpose, it is necessary to maintain a positive nitrogen balance by increasing the dietary protein intake. Several studies have shown that protein requirements of strength training athletes are higher than those of sedentary individuals [37-40]. The daily recommended protein intake is estimated to be 1.4 – 1.8 g/kg for performing resistance exercise when the intake of calories and carbohydrate is adequate [41,42] although 1.0 g/kg of protein is generally sufficient for endurance athletes excluding an elite minority [43]. It may be difficult to maintain such a high dietary protein intake, but ingestion of protein supplements can be effective. A wide variety of raw materials are utilized for the production of powdered protein supplements, and products derived from soy beans, eggs, or whey (milk protein) are commercially available. All of these products contain a good balance of essential amino acids, and often achieve an amino acid score of 100. In particular, whey protein is believed to be an ideal source for building muscles, since such protein is easily digested and absorbed, resulting in a rapid increase in the blood level of amino acids [44,45]. In addition, branched-chain amino acids and glutamine, which promote the synthesis of muscle protein, have a high content in whey protein [44,46]. Not only the amount of protein intake, but also the timing of intake are important for building muscles efficiently. Eating a meal immediately after resistance exercise may contribute to a greater increase of muscle mass compared with ingesting a meal several hours later [47-49]. Also, intake of carbohydrates with protein can accelerate the synthesis of muscle protein via the actions of insulin, which increases protein synthesis and inhibits its catabolism [50,51].

In addition, it has been reported that the intake of amino acids and peptides is beneficial. Free amino acids and peptides do not need to be digested, so rapid absorption can be expected. Amino acids are not only utilized for the synthesis of muscle protein, and some of these molecules also exert a variety of physiological effects. Attention has been focused on the effects of branched-chain amino acids (BCAAs), including valine, leucine, and isoleucine, which are known to have a relatively high content in both muscle proteins and food proteins [52]. Most amino acids are metabolized in the liver, but BCAAs are metabolized in the muscles via special processes [52,53]. BCAAs are utilized as energy substrates and their oxidation is enhanced during exercise by activation of branched-chain-α-keto acid dehydrogenase (BCKDH) complex [54]. Furthermore, BCAAs modulate muscle protein metabolism to promote the synthesis and inhibit the degradation of proteins [54-56], resulting in an anabolic effect on the muscles. Glutamine has also been reported to promote muscle growth by inhibiting protein degradation [57-59]. It is the most abundant free amino acid in muscle tissue [60] and its intake leads to an increase of myocyte volume, resulting in stimulation of muscle growth [57-59]. Glutamine is also found at relatively high concentrations in many other human tissues and has an important homeostatic role [60]. Therefore, during catabolic states such as exercise, glutamine is released from skeletal muscle into the plasma to be utilized for maintenance of the glutamine level in other tissues [61]. Arginine is a precursor of nitric oxide and creatine, and its injection promotes the secretion of growth hormone [62,63], which may lead to an increase of muscle mass and strength. Although the effect of oral arginine on protein synthesis is equivocal, recent studies have indicated that combined intake of arginine with other compounds improves exercise performance [64-66].

Various other food components have also been studied to determine their effects on muscle strength and mass. A meta-analysis of studies done between 1967 and 2001 supported the use of two supplements, creatine and β-hydroxy-β-methylbutyrate (βHMB), to augment lean body mass and strength when performing resistance exercise [67]. The human body contains more than 100 g of creatine, almost all of which is stored in the skeletal muscles as creatine phosphate. This is used to produce ATP by degradation to creatine under anaerobic conditions, so improvement of anaerobic metabolism can be expected by increasing the stores of creatine. Intake of creatine also stimulates water retention and protein synthesis [68,69]. It has been reported that the intake of ≥3 g/day of creatine increases the intramuscular content of creatine phosphate and improves endurance, especially during activities with a high power output (such as short distance running or resistance exercise), as well as improving muscle strength [70-72]. In addition, it has been reported that intake of creatine accelerates the increase of lean body mass and muscle strength during resistance training [72-74]. βHMB is a metabolite of the branched-chain amino acid leucine, and it increases muscle bulk by inhibiting the degradation of protein via an influence on the metabolism of branched-chain amino acids [75,76]. It has been reported that intake of 1.5 to 3.0 g/day of βHMB for 3 to 8 weeks achieved a greater increase of muscle mass and power compared with the intake of placebo [77-79].

## Prevention of Injury and Fatigue

Strenuous physical activity or unaccustomed exercise causes injury to the muscles, release of muscular protein, and muscle pain. The mechanism underlying delayed the muscle damage after intense physical activity is not fully understood, but it has been suggested that such delayed injury is due to an inflammatory reaction induced by phagocyte infiltration that is triggered by excessive mechanical stress [80,81], an increased intracellular Ca^2+ ^concentration [82,83], and oxidative stress [84]. There are several reports which examined whether antioxidants attenuate the muscle damage since a significant increase of oxidative products is noted in the exercised muscles and in the blood in post-exercise parallel to other parameters of delayed-onset muscle damage. Oxidative injury after acute exercise can be prevented by the intake of antioxidants, such as vitamins C and E, carotenoids, or polyphenols, not only during exercise, but also on a daily basis [84-91]. In contrast, several studies have indicated that antioxidants do not affect muscle damage and the inflammatory response caused by strenuous exercise [92-94]. One possibility on reason of the different results is that the effect of antioxidants is likely to be differences of exercise conditions, such intensity of mechanical stress and oxygen uptake. Reactive oxygen species (ROS) could be related to initiation of the muscle damage. ROS are generated from mitochondria and endothelium during exercise via elevation of the oxygen uptake of myocytes and ischemia-reperfusion process, which leads to invasion of phagocytes into the muscles after exercise via redox-sensitive inflammatory cascade. Therefore, the inflammatory response may be inhibited if ROS production during exercise is decreased just in large contribution of ROS on the initiation of muscle damage such endurance prolonged exercise not resistance exercise. Additionally, it would be better to take several antioxidants at the same time because different organelles are affected by each kind of antioxidant, such water-soluble or lipid-soluble compounds, and they can provide electrons to each other to prevent a change to pro-oxidant status.

Glucosamine and chondroitin are substances that protect the joints. Glucosamine is an amino acid synthesized in the body that is a component of synovial fluid, tendons, and ligaments in the joints. Chondroitin is mainly contained in cartilage, tendons, and the connective tissues of the skin, and plays an important role as a shock absorber due to its hygroscopic action. Supplementary oral intake of these substances is suggested to be effective for preventing or promoting recovery from osteoarthritis associated with exercise and aging [95,96] while the effect of supplementation in exercise is not clear.

There are various kinds of factors expressing fatigue condition induced by exercise such glycogen depletion and accumulation of lactic acid during exercise, and hyperactivation of sympathetic nerve in post-exercise. As mentioned above, recovery of glycogen storage in muscle is promoted by high-carbohydrate diet. At the same time, it is more effective to take a factor with an inhibitory effect on glycolysis such as citrate and consider the timing of carbohydrate intake. Also, lactate accumulation in muscle inhibits capacity of muscular contraction associated with pH decrease in muscle, which could be one of fatigue conditions. Thus, dietary supplementation regulating production or clearance lactate may be effective. Dipeptides that are abundant in skeletal muscle, carnosine and anserine, are known to have a pH-buffering effect [97]. Supplementation of these dipeptides is also possible to inhibit the decline of intramuscular pH by exercise via the buffering action of these dipeptides [98-100].

## Maintenance of Immunity

It is generally believed that moderate exercise enhances immunocompetence and is effective for the prevention of inflammatory diseases, infection, and cancer, while excessive physical activity leads to immunosuppression and an increase of inflammatory and allergic disorders [101-103]. Susceptibility to infections following excessive physical activity is ascribed to an increase in the production of immunosuppressive factors such as adrenocortical hormones and anti-inflammatory cytokines, leading to a decrease in the number and activity of circulating natural killer cells and T cells as well as a lower IgA concentration in the saliva [104]. Therefore, athletes performing high-intensity training are exposed to the risk of impaired immunocompetence. Intake of carbohydrates during prolonged exercise at submaximal intensity attenuates the increase of plasma cortisol and cytokine levels after exercise, which could lead to the inhibition of immunosuppression [105-107]. Vitamin C and vitamin E have actions that promote immunity, and are essential for T cell differentiation and for maintenance of T cell function [104,108,109]. However, there is limited evidence about the effects of vitamins supplementation on immune function in relation to exercise. Glutamine is an important energy source for lymphocytes, macrophages, and neutrophils, and is also an essential amino acid for the differentiation and growth of these cells [57,110]. Intense exercise decreases the plasma glutamine concentration and this may be related to immunosuppression [111]. Castell et al. [112] reported that athletes who ingested glutamine had a lower infection rate after a marathon compared with the placebo group. They also demonstrated that intake of glutamine resulted in an increase of the T-helper/T-suppressor cell ratio [113]. Furthermore, glutamine enhances the activity of intestinal enterobacteria and inhibits the production of cytokines involved in inflammation or immunosuppression [110].

## Conclusion

Due to a social background that includes changes of dietary habits, an aging population, and increased medical costs, people have shown a growing interest in health and have come to expect complex and diverse actions of foods. In recent years, various food factors that fulfill such requirements have been evaluated scientifically to determine whether they are any physiological effects like prevention of diseases. In the sports market, a variety of functional foods are available, but among these functional foods, some have not clearly demonstrated any efficacy and others are advertised with inappropriate and exaggerated claims, so consumers are often confused. Some of the food components described in this article should be studied further because of differing views with regard to their efficacy in different reports. Furthermore, the effectiveness of the components may differ according to gender, between individuals, and with the mode of ingestion, so that the optimum method of intake the quantity and quality of foods to be ingested, and the timing of their intake need to be established in accordance with the purpose of using each food or food component, after understanding the physiological changes by exercise. In the future, guidelines for the use and evaluation system of sports functional foods should be established with backing by clear scientific evidence related to the individual foods.

**Table 1:** Exercise and functional foods.

Physiological functions	A	B	C
Replenishment of water	Isotonic drinks	Hypotonic drinks Glycerol	
Improvement of endurance	High-carbohydrate Citric acid	Arginine Caffeine Carnitine	Capsaicine
Enhancement of muscle strength	Protein BCAA Creatine β-HMB	Glutamine	Arginine
Prevention of muscle/joint injuries or fatigue	High-carbohydrate Citric acid	Vitamins C and E Carotenoids, Flavonoids Carnosine, Anserine	Glucosamine Chondroitin
Prevention of a decrease in immunocompetence	Carbohydrate	Vitamins C and E Glutamine	

A: The factors in this group has been shown adequate scientific evidence.

B: The factors in this group has been shown suggestive evidence.

C: The factors in this group has been shown no evidence while possible to effective.
